# Enhanced recovery from fulminant myocarditis by treatment with the combined use of the Impella left ventricular assist device with extracorporeal membrane oxygenation: a case series

**DOI:** 10.1186/s40981-022-00502-x

**Published:** 2022-02-27

**Authors:** Hideyuki Nandate, Tasuku Nishihara, Yukihiro Nakata, Taisuke Hamada, Yasushi Takasaki, Toshihiro Yorozuya

**Affiliations:** Department of Anesthesia and Perioperative Medicine, Ehime University Graduate School of Medicine, Toon, Ehime Japan

**Keywords:** Fulminant myocarditis, Impella, V-A ECMO, Cardiogenic shock, LV unloading

## Abstract

**Background:**

We experienced two adult cases of fulminant myocarditis with severe cardiogenic shock where Impella left ventricular assist device [left ventricle (LV)-Impella] was concomitantly used with venoarterial extracorporeal membrane oxygenation (V-A ECMO).

**Case presentation:**

A 67-year-old man and a 49-year-old man with fulminant myocarditis were transferred to our hospital with mechanical support of V-A ECMO and IABP. Impella 5.0 and Impella CP were implanted 21 h and 17 h after establishing V-A ECMO for each case. Within 1 week, the patients’ LV function progressively improved. Then the Impellas were withdrawn after discontinuing V-A ECMO. They were discharged from the intensive care unit within the following 8 days.

**Conclusions:**

The optimal introducing timing of LV-Impella is not currently precise. However, this case report suggests that the initiation of LV-Impella within at least 24 h after establishing V-A ECMO may be acceptable for the recovery of cardiac function.

## Background

Fulminant myocarditis is a fatal inflammatory disease of the myocardium that is complicated with refractory cardiogenic shock [1]. This condition frequently requires advanced treatment with mechanical support, such as venoarterial extracorporeal membrane oxygenation (V-A ECMO) and intra-aortic balloon pumping (IABP) [2]. Recent clinical reports have described that the use of the Impella left ventricular assist device [left ventricle (LV)-Impella], which can directly cause LV unloading, is a practical method of mechanical support for patients with fulminant myocarditis [3–7]. We here experienced two adult cases of fulminant myocarditis with severe cardiogenic shock that was successfully treated with concomitant use of LV-Impella on V-A ECMO.

## Case presentation

### Case 1

A 67-year-old man with fulminant myocarditis was transferred to our university hospital the day after he experienced cardiac arrest in a regional hospital. His cardiopulmonary resuscitation had been thriving under the mechanical support of peripheral V-A ECMO combined with IABP. On arrival at our hospital, laboratory data showed acute deterioration of the liver and renal function (serum aspartate aminotransferase [AST], 704 IU/L; serum alanine aminotransferase [ALT], 530 IU/L; serum creatinine, 2.09 mg/dL). Severe metabolic acidosis of pH 7.211 was present, with a higher level of lactate (66 mg/dL), and transthoracic echocardiography (TTE) showed that the global wall motion of the LV was severely depressed, with an estimated ejection fraction (eEF) of approximately 10%. The serum concentration of cardiac troponin I was 10,089.3 pg/mL. Endocardial biopsy revealed histopathologic findings consistent with fulminant myocarditis.

**Fig. 1:**
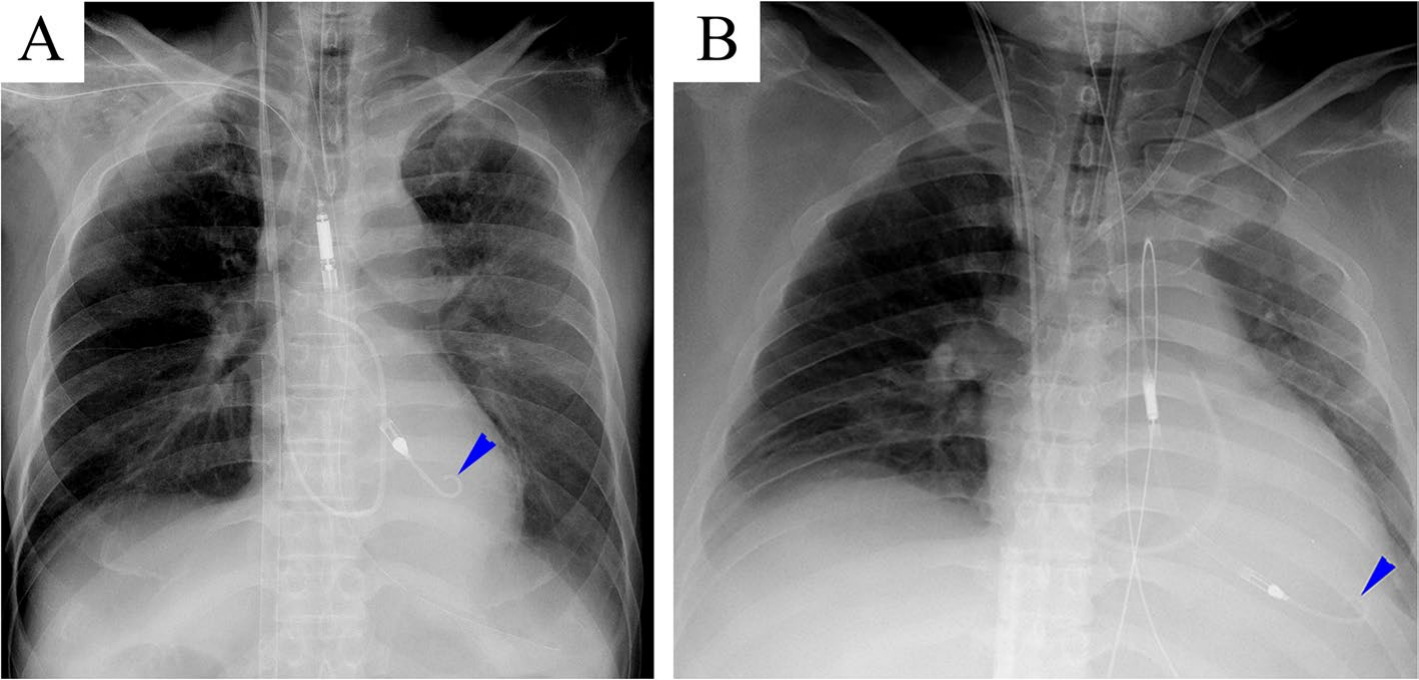
Supine chest x-rays in two cases. **A** A supine chest x-ray in a 67-year-old man who presented with fulminant myocarditis on the left side. The blue arrowhead indicates the tip of the Impella 5.0 indwelled via a vascular prosthesis surgically anastomosed to the right subclavian artery. **B** A supine chest x-ray in a 49-year-old man presented with fulminant myocarditis on the right side. The blue arrowhead indicates the tip of the Impella CP indwelled percutaneously via the left femoral artery

**Fig. 2:**
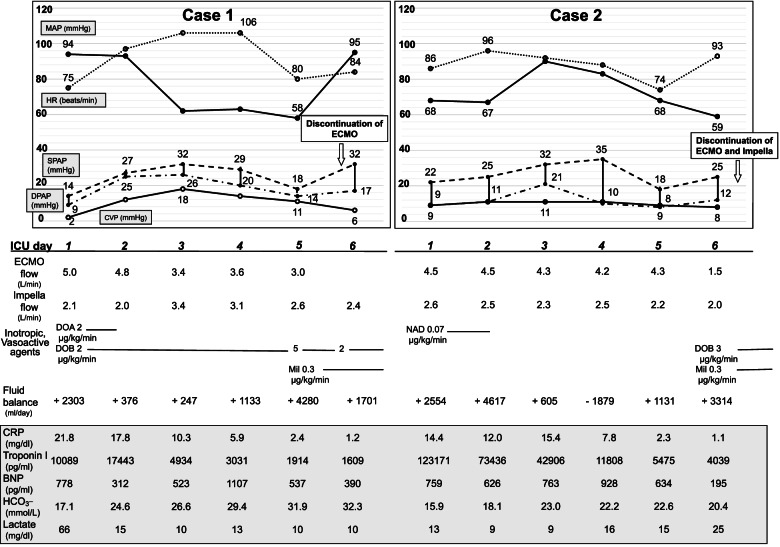
Clinical course chart in two cases. Case 1 and 2 were presented on the left and right sides, respectively. MAP (  ), mean arterial pressure; HR (  ), heart rate; SPAP (  ), systolic pulmonary arterial pressure; DPAP (  ), diastolic pulmonary arterial pressure; CVP (  ), central venous pressure; CVP, central venous pressure; CRP, C-reactive protein; BNP, brain natriuretic peptide; DOA, dopamine; DOB, dobutamine; NAD, noradrenaline; Mil, milrinone

Because the use of the Impella CP was not commercially available in Japan at that time, we implanted the Impella 5.0 via a vascular prosthesis surgically anastomosed to the right subclavian artery under fluoroscopic guidance (Fig. 1A) and removed the IABP. After working the Impella 5.0, the diastolic pulmonary arterial pressure decreased from over 25 mmHg to below 15 mmHg in the operating room. Twenty-one hours had passed after the establishment of V-A ECMO. After the initiation of combined treatment with the Impella 5.0 on V-A ECMO, with the flow rate initially set as 2.0 L/min and 5.0 L/min, respectively, metabolic acidosis improved to pH 7.440, with a further decrease in the serum level of lactate of 19 mg/dL. Additionally, we supplemented anti-inflammatory treatment with a massive dose of γ-globulin administered at a rate of 5 g/h for 48 h along with steroids. Methylprednisolone 1000 mg a day was administered for 2 days, and the subscribed dose was gradually tapered off. A low dose of dobutamine such as 2 μg/kg/min was continuously infused under mechanical support with the combination of V-A ECMO and Impella 5.0 (Fig. 2). On the fifth day, the patient’s LV function progressively improved, with an eEF of approximately 60% under inotropic support. Dobutamine and milrinone were infused at a rate of 2 μg/kg/min and 0.3 μg/kg/min, respectively. Consequently, we discontinued V-A ECMO, and on the next day, we also withdrew the Impella 5.0. However, it took an additional 2 days for pulmonary congestion to improve sufficiently. On the seventh day, spontaneous respiration without a tracheal tube recovered under high-flow oxygen therapy via nasal cannula. After his discharge from the intensive care unit (ICU) on the 14th day, the patient performed active rehabilitation for the remaining period of hospitalization because of unusual muscle weakness in the bilateral upper and lower extremities, which was diagnosed as critical illness neuropathy. On the 30th day, he was transferred to the original hospital for continuation of rehabilitation.

### Case 2

A 49-year-old man with fulminant myocarditis was transferred from a regional hospital to our university hospital. One day before, the patient had developed severe circulatory disturbance with refractory ventricular fibrillation that required mechanical support with peripheral V-A ECMO combined with IABP. On arrival at our hospital, laboratory data showed serum concentrations of AST, ALT, creatinine, and cardiac troponin I of 634 IU/L, 169 IU/L, 3.11 mg/dL, and 123,171.1 pg/mL, respectively. TTE showed that the global wall motion of LV was severely depressed, with an eEF of approximately 13%. At 17 h later from the establishment of V-A ECMO, we implanted Impella CP percutaneously via a left femoral artery after the removal of the IABP under fluoroscopic guidance (Fig. 1B). An endocardial biopsy suggested suspicion of necrotizing eosinophilic myocarditis. We initially set the flow rate of the Impella CP and V-A ECMO as 2.0 L/min and 4.5 L/min, respectively. Because of sustained oliguria, we introduced renal replacement therapy with a continuous venovenous hemodiafiltration (CHDF). Additionally, we administered a massive dose of γ-globulin and methylprednisolone in the same manner as in case 1. An infusion of inotropic and vasoactive agents was free until one day before the termination of mechanical support (Fig 2). On the seventh day, we noted a progressive improvement of LV function with an eEF of approximately 65% under inotropic support. Dobutamine and milrinone were infused at a rate of 3.3 μg/kg/min and 0.26 μg/kg/min, respectively. Contact between the tip of the Impella CP and the ventricular wall probably induced frequent ventricular ectopic beats because of the reduced volume of the LV chamber. We discontinued V-A ECMO and withdrew the Impella CP. Because of persistent pulmonary congestion, we continued mechanical ventilation using high positive end-expiratory pressure with a large volume of filtration by employing CHDF. On the 12th day, the patient began spontaneously breathing under high-flow oxygen therapy via nasal cannula after the removal of the tracheal tube. After his discharge from the ICU with termination of CHDF on the 15th day, psychological dysfunction, such as delirium, remained, which might have been caused partially by initial hypoxic brain damage. On the 30th day, the patient was transferred to the original hospital for mental care and rehabilitation.

## Discussion

In these two patients, the initial mechanical support consisted of a combination of V-A ECMO with IABP for refractory cardiogenic shock. Although V-A ECMO is helpful for restoring a disturbance in systemic circulation, the prognosis of patients with LV failure is reportedly not as good as we expect, despite advanced mechanical support with V-A ECMO [8]. With it being likely that the endodiastolic LV pressure in these patients was remarkably elevated enough to induce pulmonary congestion, which indicated that an additional therapeutic option was required for LV decompression, we suggest that the main reason for this outcome is that the V-A ECMO increases the afterload to the damaged LV, leading to a further rise in LV wall stress and a subsequent reduction of subendocardial coronary flow [9].

We inserted the LV-Impella equipped with a microaxial flow pump directly across the aortic valve into the LV chamber. This served to unload the LV by venting out a certain quantity of blood from the LV cavity and delivering it into the ascending aorta, which made a retrograde flow of V-A ECMO reduced. In addition, as shown in the clinical course chart, we could reduce or eliminate the use of inotropic and vasoactive agents, which might increase wall stress and oxygen consumption in the injured myocardium under mechanical support with the combination of V-A ECMO and LV-Impella. A previous retrospective study using a multicenter cohort compared the outcome of treatment between V-A ECMO alone and a combination of V-A ECMO and LV-Impella in 157 patients suffering from severe cardiogenic shock, including five patients with fulminant myocarditis, of whom 123 (78%) received V-A ECMO alone and 34 (22%) received a combination of V-A ECMO and LV-Impella, respectively [10]. Using propensity score matching, the study showed that the combined treatment group had a lower rate of hospital mortality (47% vs. 80%, *P* < 0.001) and a higher rate of successful transition to either recovery or further therapy (68% vs. 28%, *P* < 0.001) [10].

A more extensive retrospective study in a multicenter registry enrolled 34 patients with fulminant myocarditis employing LV-Impella [11]. Despite that study being retrospective and enrolling a small number of patients so it might have had limited information regarding the mechanical support for fulminant myocarditis, the study suggested the safety and effectiveness of LV-Impella based on the survival rate of 62% (21 patients, including a successful recovery of 15 patients) in the setting of cardiogenic shock [11]. In addition, although a multicenter cohort study in 686 cardiogenic shock patients treated with V-A ECMO showed that an introduction of LV-Impella < 2 h after V-A ECMO was associated with lower mortality risk. However, the optimal timing of unloading with LV-Impella was still unclear [12]. Therefore, several questions remain to be answered, including the optimal timing of initiation of LV-Impella and the ideal duration of support necessary for recovery or to obtain a bridge to further therapy.

Myocarditis is an inflammatory disease, and in affected myocardial tissue, active inflammation such as cellular infiltrates develops, resulting in damage to cardiomyocytes. It is possible that early unloading to the affected LV during the active inflammatory phase may have a positive therapeutic effect against the progression of inflammatory insult, which is different from cases of ischemic LV dysfunction [9]. In general, the use of the LV-Impella on V-A ECMO as soon as cardiogenic shock emerges, if possible, may result in a better prognosis. Nishihara et al. reported a case of thrombosis in the ascending aorta during V-A ECMO management and suggested combined use of LV-Impella from as soon as possible especially in case of very low cardiac function [13].

However, in the cases reported here, we were unable to introduce the LV-Impella soon after the sudden development of cardiogenic shock because it took several hours to transfer the patients to our university hospital. Although there was a time delay of several hours until the initiation of LV-Impella support, these two patients achieved a full recovery of LV function within approximately 6 days after the start of LV unloading. Thus, a delay of several hours until the introduction of the LV-Impella might be acceptable, with the expectation that an almost full recovery of LV function can be achieved in a short duration, such as 6 days. Therefore, while the decision to initiate the LV-Impella should be made within at least 24 h from the establishment of V-A ECMO, once the patient is stabilized with cardiogenic shock by mechanical support with V-A ECMO, physicians have time to decide whether the LV-Impella is necessary. Subsequently, further accumulation of cases with fulminant myocarditis is required to clarify our suggestion for treatment with LV-Impella.

In these two patients, mechanical support using the LV-Impella was implemented in addition to V-A ECMO within 24 h after the development of severe cardiogenic shock. They had a full recovery of LV function within approximately 6 days after LV unloading.

## Conclusion

The optimal introducing timing of LV-Impella is not currently precise. However, this case report suggests that initiation of LV-Impella within at least 24 h after the establishment of V-A ECMO may be acceptable for the recovery of cardiac function.

## Data Availability

All data generated or analyzed during this study are included in this published article.

## Abbreviations

ALT: Serum alanine aminotransferase
AST: Serum aspartate aminotransferase
CHDF: Continuous venovenous hemodiafiltration
eEF: Estimated ejection fraction
IABP: Intra-aortic balloon pumping
ICU: Intensive care unit
LV: Left ventricle
LV-Impella: Impella left ventricular assist device
TTE: Transthoracic echocardiography
V-A ECMO: Venoarterial extracorporeal membrane oxygenation
